# Mississippi Valley Association of Dental Surgeries—Thirty-Seventh Annual Meeting

**Published:** 1881-04

**Authors:** W. H. Sillito


### MISSISSIPPI VALLEY ASSOCIATION OF DENTAL SURGEONS—THIRTY-SEVENTH ANNUAL MEETING.

WEDNESDAY, THURSDAY AND FRIDAY, MARCH 3RD, 4TH AND 5TH, 1881.

### FIRST DAY.—MORNING SESSION.

The Association met in the Hall of the Ohio Dental College, College Street, Cincinnati, at 10-30 o'clock. The president, Dr. H. M. Reid, in the chair.

Prayer was offered by Dr. James Leslie.

The minutes of the last annual meeting were read, and, on motion, approved.

The chairman of the executive committee, Dr. G. W. Keely, reported the following list of subjects for discussion:

1. Alveolar abscess, and treatment.
2. Treatment of superficial caries of the teeth.
3. To what extent does vitality resist the encroachment of dental caries?
4. Removal of the first permanent molars; when indicated?
5. Pathology and treatment of exposed pulps.
6. Diseases of the antrum, and treatment.
7. To what extent should other materials than gold be used in filling teeth?
8. Artificial dentures.

On motion, adopted.

The President requested members to pay their dues to the Secretary during the absence of the Treasurer.

W. H. Sillito was elected Secretary pro tem., the Recording Secretary, Dr. Betty, being engaged to report the discussions of the meeting.

The Treasurer made the following report:

| | | |
|---|---|---|
| Balance on hand | 28.75 | |
| Received dues, 1880 | 45.00 | |
| " initiation fees, 1880 | 18.00 | |
| | $91.75 | |
| Paid Recording Secretary, 1880 | 15.00 | |
| " Janitor, 1880 | 6.00 | |
| " Executive Committee for printing | 4.50 | |
| " Corresponding Secretary for postage | 2.50 | 28.00 |
| Cash on hand | | 63.75 |
| | | $91.75 |

(Signed) J. G. CAMERON, Treas.

On motion, received, and referred to Drs. F. A. Hunter and G. W. Keely as a committee to audit.

On motion, the hours of meeting were set as follows:

1st Day—Morning session to adjourn at noon.
Afternoon " 2 to 4 o'clock.
Night " 7½ to adjournment.
2nd Day—Morning " 9 to 12 o'clock.
Afternoon " 2 to 5 "
3rd Day—Morning " 9 to 12 "

The discussion of the first subject, alveolar abscess and treatment, was opened by Dr. J. Taft, and continued by Drs. F. A. Hunter, W. D. Kempton and James Taylor.

Adjourned.

AFTERNOON SESSION.

The meeting was called to order at 2 30 o'clock by President Reid. Minutes of morning session read, and, on motion, approved.

The discussion of the first subject was resumed by Drs. Kempton, Cassidy, Morrill, Jay, G. W. Keely, J. Taft, H. A. Smith and W. S. How.

On motion, the first and second subjects were passed.

The hour for adjournment having arrived, Dr. J. Taft moved that the session be extended one-half hour. Carried; but, upon re-consideration, the motion was annulled

Adjourned.

NIGHT SESSION.

The Association came to order at 7-30 o'clock. President Reid in the chair.

Minutes of the previous session were read, and, on motion, approved.

The consideration of the third subject—To what extent does vitality resist the encroachment of dental caries?—was now in order.

Dr. A. Berry opened the discussion, followed by Drs. Sage, Kempton, Jay, J. Taft and Leslie.

On motion, subject passed.

Adjourned.

SECOND DAY—MORNING SESSION.

Meeting called to order at 10 o'clock by President Reid.

The minutes of last night's session were read and approved.

Dr. James Taylor proposed Dr. J. T. Irwin, of Cincinnati, for membership.

On motion, the Secretary was instructed to cast a ballot electing Dr. Irwin.

Dr. F. A. Hunter opened the discussion of the fourth subject—Removal of first permanent molars, when indicated?—which was continued by Drs. J. Taft, Berry, James Taylor, Osmond, G. W. Keely, J. F. Dennis, Leslie, H. A. Smith, Jay and Porre.

On motion, the thanks of the Association were tendered Dr. Keely for his complete presentation of cases, by models and paintings illustrating the subject.

On motion, the subject was passed.

Adjourned.

AFTERNOON SESSION.

The Society was called to order at 2-30 o'clock by the President, Dr. Reid.

Minutes of morning session read and adopted.

On motion, the fifth and sixth subjects were laid over, and the seventh and eighth taken up.

The discussion of the seventh—To what extent should other materials than gold be used in filling teeth?—was opened by Dr. James Taylor, and participated in by Drs. Ulrey, McCampbell, Jay, Rawls, and J. Taft.

On motion, the President appointed Drs. James Taylor, J. R. Clayton and G. W. Keely as a committee to report the sense of the meeting in regard to the death of Dr. M. Rogers.

They did so, as follows:

In reporting on the decease of Dr. M. Rogers, which occurred July 24th, 1880, at the age of 84, we feel that God has taken from us one of the founders of this Society—one who, by his high standing, and his medical qualifications, had always added lustre to the profession he had chosen for his life work. He might, perhaps, justly be regarded as the first permanently located dentist of this city. Here he labored during the whole of his professional life. Here he located to practice medicine; but fortunatly some troublesome cases of dental origin turned his special attention to the need of scientific dental surgery; hence he soon became exclusively confined to this specialty, and for its ultimate good and elevation he assiduously labored. We find him connected with the organization of this Association; and when the Dental College was organized he was appointed to the chair of pathology and therapeutics; and, wherever placed, his special qualifications made him an efficient co-laborer.

Therefore, while thankful for his great services, we yet with sad hearts bow to the dispensation of providence in his removal from us. That his family, to whom we offer our deepest sympathy, be notified of our action. (Signed) JAMES TAYLOR, Chairman.

On motion, adopted, and ordered spread upon the minutes.

Dr. H. A. Smith read a paper on civil malpractice in its relation to dentistry.

On motion, the paper was requested for publication, and referred to the proper committee.

Remarks on the subject of the paper read by Prof. Smith were made by Drs. J. Taft, G. W. Keely, Rawls, James Taylor, Cassidy, Sillito, Berry and Sage.

On motion, the Secretary cast a ballot electing to membership Drs. J. R. Callahan, of Hillsboro, O., and J. M. Clyde, Covington, Kentucky.

On motion, Dr. J. G. Cameron was allowed to make a statement in regard to a case which occurred in his practice.

Adjourned.

THIRD DAY—MORNING SESSION.

The Society came to order at 9-30 o'clock. President Reid in the chair.

The minutes of the previous session were read, and, on motion, adopted.

The seventh subject was passed.

On motion, the election of officers was set for 10-30 A. M.

On motion, the fifth subject—pathology and treatment of exposed pulps—was taken up and discussed by Drs. J. Taft, Van Antwerp, Fletcher and Osmond

By order of the Association the Secretary cast a ballot electing to membership Drs. S. B. Cook, of Sweetwater, Tenn.; T. Y. Cooper, of Owingsville, Ky.; G. S. Junkerman, of Cincinnati; and C. R. Sabin, of Troy, O.

The time for the election of officers having arrived, the Association proceeded to ballot, with the following result:

President—J. W. Jay, Richmond, Ind.

First Vice-President—Wm. Van Antwerp, Mt. Sterling, Ky.

Second Vice-President—C. I. Keely, Hamilton, O.

Recording Secretary—N. S. Hoff, Cincinnati.

Corresponding Secretary—H. L. Moore, Cincinnati.

Treasurer—F. A. Hunter, Cincinnati.

Drs. C. R. Taft and G. W. Keely were appointed a Committee to conduct the President-elect, Dr. Jay to the Chair; the latter briefly returning thanks for the honor conferred by his election to preside over the oldest dental society in the world.

On motion, thanks were tendered the retiring officers for efficient performance of their duties.

On motion, the President appointed a Committee, consisting of Drs. Cassidy and Hunter, to select delegates to the meeting of the American Dental Association, in New York City, next August.

They reported the following persons for delegates:

Otto Arnold, Ironton, Ohio.
T. S. Brown, Gallipolis, O.
E. G. Betty, Cincinnati.
T. Y. Cooper, Owingsville, Kentucky.
J. R. Callahan, Hillsboro, O.

S. B. Cook, Sweetwater, Tennessee.
J. M. Clyde, Covington, Covington, Ky.
J. G. Cameron, Cincinnati.
M. H. Fletcher, Cincinnati.
O. N. Heise, Cincinnati.
J. T. Irwin, Cincinnati.
J. W. Jay, Richmond, Indiana.
C. I. Keely, Hamilton, O.
H. L. Moore, Cincinnati.
H. M. Reid, Cincinnati.
A. G. Rose, Cincinnati.
C. R. Sabin, Troy, Ohio.
A. Taft, Dayton, O.

On motion, adopted.

The President appointed the following Standing Committees for the ensuing year:

Executive—J. S. Cassidy.
M. H. Fletcher.
N. S. Hoff.

Membership—J. G. Cameron.
T. S. Brown.
C. I. Keely.

Publication—Jas. Taylor.
Jas. Leslie.
E. G. Betty.

Ethics—H. A. Smith.
G. W. Keely.
C. R. Taft.

On motion, six dollars were appropriated to pay the janitor of the College for services.

On motion, fifteen dollars were appropriated to pay the Recording Secretary.

Adjourned to meet at the same place, the first Wednesday in March, 1882.

W. H. SILLITO,
*Recording Secretary, pro tem.*

